# Mobile Personal Health Care System for Noninvasive, Pervasive, and Continuous Blood Pressure Monitoring: Development and Usability Study

**DOI:** 10.2196/18012

**Published:** 2020-07-20

**Authors:** Luis J Mena, Vanessa G Félix, Rodolfo Ostos, Armando J González, Rafael Martínez-Peláez, Jesus D Melgarejo, Gladys E Maestre

**Affiliations:** 1 Academic Unit of Computing, Master Program in Applied Sciences Universidad Politecnica de Sinaloa Mazatlan Mexico; 2 Faculty of Information Technology Universidad de la Salle Bajío Leon Mexico; 3 Research Unit Hypertension and Cardiovascular Epidemiology KU Leuven Department of Cardiovascular Sciences University of Leuven Leuven Belgium; 4 Departments of Neurosciences and Human Genetics, and Rio Grande Valley Alzheimer´s Disease Resource Center for Minority Aging Research University of Texas Rio Grande Valley Brownsville, TX United States

**Keywords:** mHealth, photoplethysmography, blood pressure monitoring, hypertension

## Abstract

**Background:**

Smartphone-based blood pressure (BP) monitoring using photoplethysmography (PPG) technology has emerged as a promising approach to empower users with self-monitoring for effective diagnosis and control of hypertension.

**Objective:**

This study aimed to develop a mobile personal health care system for noninvasive, pervasive, and continuous estimation of BP level and variability, which is user friendly for elderly people.

**Methods:**

The proposed approach was integrated by a self-designed cuffless, calibration-free, wireless, and wearable PPG-only sensor and a native purposely designed smartphone app using multilayer perceptron machine learning techniques from raw signals. We performed a development and usability study with three older adults (mean age 61.3 years, SD 1.5 years; 66% women) to test the usability and accuracy of the smartphone-based BP monitor.

**Results:**

The employed artificial neural network model had good average accuracy (>90%) and very strong correlation (>0.90) (*P*<.001) for predicting the reference BP values of our validation sample (n=150). Bland-Altman plots showed that most of the errors for BP prediction were less than 10 mmHg. However, according to the Association for the Advancement of Medical Instrumentation and British Hypertension Society standards, only diastolic blood pressure prediction met the clinically accepted accuracy thresholds.

**Conclusions:**

With further development and validation, the proposed system could provide a cost-effective strategy to improve the quality and coverage of health care, particularly in rural zones, areas lacking physicians, and areas with solitary elderly populations.

## Introduction

### Background

Although hypertension is the most important modifiable risk factor for global disability-adjusted life-years among diseases, injuries, and risk factors [1], its prevalence continues to increase globally. Currently, an estimated 1.13 billion people worldwide have hypertension (blood pressure [BP] ≥140/90 mmHg) [2]. It is known as a “silent killer,” as its signs and symptoms can be very subtle and slow until after key organs are severely affected. To identify BP dysregulation and ascertain if treatment or lifestyle modifications are appropriate for control, BP needs to be measured frequently and in real-life conditions. Hypertension prevalence rates are usually based on clinic BP measures, but these can exclude a substantial number of people who have “masked” hypertension (normotensive by clinical measurement, but hypertensive by ambulatory or home BP readings) [3], which confers cardiovascular risk similar to that of sustained hypertension [4]. Therefore, the development and implementation of new strategies to stratify population-level risk and identify people who are treated but still have uncontrolled hypertension or have “masked” hypertension are desperately needed.

Ambulatory BP monitoring is the noninvasive gold standard for the clinical diagnosis of BP dysregulation, as it records BP over a period of 24 hours or more using automated upper-arm cuff oscillometric devices during normal daily activities, thereby providing a much more complete and representative picture of BP patterns than traditional single clinic-based measurement [5]. Continuous BP monitoring records can provide an estimate of the true BP level, the circadian BP profile, and information on inherent BP variability, which is considered a potential independent predictor of cardiovascular events or complications [6-8]. Nevertheless, the periodic inflation of the cuff to register BP readings is associated with moderate discomfort or pain and severe restriction in everyday activities during the monitoring period [9].

The use of noninvasive and cuffless BP monitors has emerged as a more comfortable and user-friendly alternative to estimate BP measurement [10]. The adoption of photoplethysmography (PPG) technology has led to the development of small, inexpensive, and wearable optoelectronic sensors for long-term BP monitoring [11-13]. The underlying principle of the PPG method is based on illumination of the skin by a light-emitting diode (LED) and measurement of the amount of light that is absorbed or reflected from living tissue using a luminosity sensor. Since the tissue changes its vascular tone according to the volume of blood pumped by the heart during each cardiac cycle, the PPG signal can detect changes in volumetric blood flow by the photoelectric technique [11,13]. Thus, variations in volume and distension of the arteries can relate to the heart’s systolic and diastolic periods to estimate BP.

Most PPG-based BP monitors have been developed through synchronicity-dependent approaches that measure the PPG signal simultaneously with another biosignal or two PPG signals from different body sites. This type of approach requires two sensors to calculate the time that a single heartbeat pulse takes to travel from the heart to a peripheral location or from one arterial site to another. Generally, an initial or periodic calibration step using an additional BP monitor as a reference is required to obtain a more reliable BP measurement [14-17]. Another simple and less obtrusive approach uses a single PPG sensor. BP is estimated by extracting time and frequency features derived from the PPG waveform to examine the similarity between PPG and arterial BP morphologies or directly using the raw PPG signal [18]. However, PPG-only approaches need more robust methods to accurately describe the inherent but not well-established relationship between PPG signal variations and BP changes [11,19].

Parallel advances in sensing technologies combined with the growth of mobile computing and wireless communication are shifting health care from traditional clinic monitoring to real-time personal monitoring. The development and application of mobile personal health care systems could improve quality and coverage, while reducing costs through early detection [20,21]. Thus, the use of a smartphone equipped with built-in or external sensors is considered a promising approach to empower users with self-monitoring for the effective diagnosis and control of hypertension and to facilitate timely patient and health care provider communication for medical feedback and clinical support [22-27]. Underserved populations (mainly elderly people living in developing countries) will benefit from mobile technology [28,29].

On the other hand, with the increasing advancement and popularity of machine learning methods, novel ways to improve BP estimation from a single PPG signal without any additional sensor have emerged [13,19]. Given that light emitted by LEDs can penetrate an area that involves skin, arteries, veins, blood, bone, and other tissues, optical absorption changes detected by a PPG sensor represent a complex mixture of pulsatile and nonpulsatile blood flow components [13,30]. Therefore, heuristic modeling based on advanced artificial neural networks (ANNs) using nonlinear regression could aid in dealing with confounding factors and improve the result of BP estimation from feature extraction or raw PPG signals [19,31-34].

### Recent Work

We provide an overview of recent work on smartphone-based BP monitors using single or combined PPG approaches, whose estimates were compared with measures recorded via a reference BP device (Table 1) [35-42].

**Table 1:** Overview of smartphone-based blood pressure monitors using single or combined photoplethysmography approaches.

Smartphone PPG^a^ approach	Correlation coefficient		Error bias (mmHg), mean (SD)			BP^b^ measures		Reference BP device
	SBP^c^	DBP^d^	SBP	DBP				
Chandrasekaran et al [35]	NR^e^	NR	2.45^f^	1.71^f^	500		Mercury sphygmomanometer	
Plante et al [36]	0.44^g^	0.41^g^	12.40 (10.50)^h^	10.10 (8.10)^h^	101		Omron HEM-907	
Wang et al [37]	NR	0.81^i^	NR	6.70^j^	196		Microlife BP3NA1-1x	
Chandrasekhar et al [38]	0.76^i^	0.79^i^	3.30 (8.80)^f^	−5.60 (7.70)^f^	32		Omron BP7650N	
Chandrasekhar et al [39]	0.79^i^	0.78^i^	−4.00 (11.40)^f^	−9.40 (9.70)^f^	18		Omron BP786	
Raichle et al [40]	0.40^g^	NR	5.00 (14.50)^h^	NR	96		Omron HBP-1300	
Dey et al [41]	NR	NR	6.90 (9.00)^h^	5.00 (6.10)^h^	205		Mercury sphygmomanometer	
Luo et al [42]	0.67^i^	0.47^i^	0.39 (7.30)^f^	−0.20 (6.00)^f^	1328		CNAP Monitor 500	

^a^PPG: photoplethysmogram.

^b^BP: blood pressure.

^c^SBP: systolic blood pressure.

^d^DBP: diastolic blood pressure.

^e^NR: not reported.

^f^Mean error.

^g^Spearman correlation.

^h^Mean absolute error.

^i^Pearson correlation.

^j^Root mean square error.

#### PPG-Combined Approaches

Chandrasekaran et al [35] made the earliest attempts to monitor BP changes using an external microphone and smartphone camera for recording heart sounds and corresponding finger pulse. From preprocessed phonocardiography and PPG signals, they calculated the vascular transit time [43] (transmission delay of blood from the heart to a body peripheral point for one heartbeat) to estimate BP values. Approach accuracy was assessed by separately measuring BP using a commercial sphygmomanometer. However, the data collection procedure and correlation analysis with BP reference measures were not reported.

A validation study of this method was performed by Plante et al [36] using a commercial iOS app to estimate BP from PPG and phonocardiography signals collected with a smartphone camera and microphone. Simultaneous BP measures were taken using a validated oscillometric device and smartphone to calculate the mean and SD of absolute differences, correlation coefficient, and British Hypertension Society (BHS) accuracy grade [44] between reference and test values. The findings indicated a highly inaccurate BP estimation by showing weak correlation, the lowest possible BHS score, and 78% misclassification of hypertensive measures.

In 2018, Wang et al [37] developed a smartphone BP monitoring app using a built-in accelerometer and camera to capture vibration caused by heartbeats and fingertip pulse. Only diastolic BP (DBP) measures were estimated by calculating pulse transit time [17] (time taken by a heart pulse to travel between two arterial sites), using seismocardiography and PPG signals. DBP estimated with initial calibration showed very strong correlation and adequate average error against reference values measured simultaneously with a commercial oscillometric device.

Additionally, in 2018, Chandrasekhar et al [38] developed a calibration-free BP measurement smartphone device based on an extension of the oscillometric principle. The approach involved external PPG and force sensors to detect blood flow and applied pressure from a finger, and an Android app to calculate and display BP estimates. Comparison with reference values obtained by a standard oscillometric device showed a strong correlation and error range close to the limits established by the Association for the Advancement of Medical Instrumentation (AAMI) [45].

Thereafter, the same authors [45] developed an iOS app to estimate BP via a similar method, but using the smartphone camera to detect blood volume oscillations and a strain gauge array under the screen to measure finger pressure [39]. The assessment of accuracy also demonstrated high correlation and good agreement in terms of error bias and precision with respect to standard reference measures.

#### PPG-Only Approaches

In 2018, Raichle et al [40] conducted a study to assess the performance of an iOS app estimating systolic BP (SBP) via PPG signals recorded by a smartphone camera from the finger. The estimation algorithm determined BP based on morphology and frequency analysis of PPG signals. Validation results reported a moderate correlation, an adequate mean absolute error but with high SD, and the worst BHS accuracy grade in comparison with reference SBP measured using an oscillometric device.

In the same year, Dey et al [41] developed an ensemble of BP prediction models based on PPG feature extraction and values of demographic and physiological profiles. PPG signals were acquired using a heart rate sensor embedded in a smartphone, and an Android app was used to display BP estimates. A machine learning approach using the Lasso regression model [46] was adopted for initial DBP estimation, and subsequently, this output was used as feedback to derive SBP. The combined model of PPG features and demographic and physiological partitioning showed adequate absolute error and precision with respect to reference BP measured using a mercury-based cuff device.

More recently, Luo et al [42] proposed a BP monitoring approach using a variant of the remote PPG technique to detect facial blood flow changes from videos captured with a smartphone camera, and a multilayer perceptron machine learning algorithm was used for BP estimation. The prediction model used orthogonal eigenvectors as input by applying principal component analysis on features extracted from facial blood flow signals, metafeatures for imaging condition normalization, environmental temperature variations, and individual physical characteristics. Assessment against reference measures from a continuous finger BP monitor indicated moderate and strong correlations for DBP and SBP, and high BP prediction accuracy and precision according to the AAMI standard [45].

### Goal of This Study

In this study, we address these issues by developing a mobile personal health care system for noninvasive, pervasive, and continuous BP monitoring, with the goal of improving the early diagnosis and control of hypertension and identifying a potential cardiovascular predictor (abnormal BP variability). The proposed approach is integrated by a self-designed cuffless, calibration-free, wireless, and wearable PPG-only sensor and a native smartphone app designed to be user friendly for elderly people, which is based on machine learning techniques for BP level and variability estimation from raw PPG signals.

## Methods

### Brief Description

This section provides an overview of our approach, which is based on the development of a PPG-only sensor to acquire and transfer raw signals to a smartphone and a mobile app using multilayer perceptron machine learning techniques to estimate BP level and variability.

### Approach

**Figure 1:**
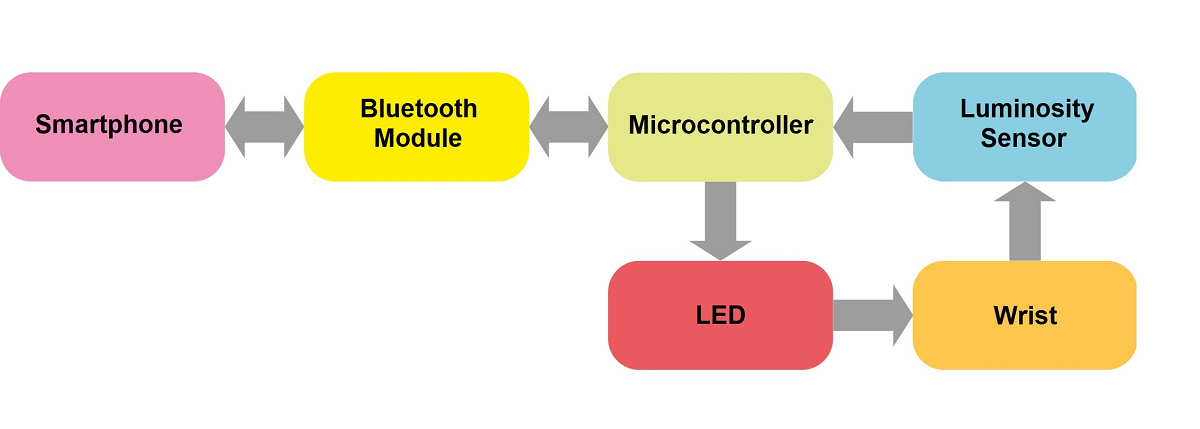
Block diagram of the blood pressure estimation approach.

The mobile personal health care system consists of a wearable sensor based on a portable microcontroller provided with Bluetooth transmission and PPG technology to detect light absorption changes from the wrist and a smartphone using ANN algorithms to estimate BP parameters. Communication between the PPG sensor and smartphone occurs via a master-slave scheme controlled by the mobile device. Therefore, all BP estimations are started by the master device (smartphone), which receives responses from the slave device (PPG sensor).The basic block diagram of the proposed approach is shown in Figure 1.

### PPG Sensor

**Figure 2:**
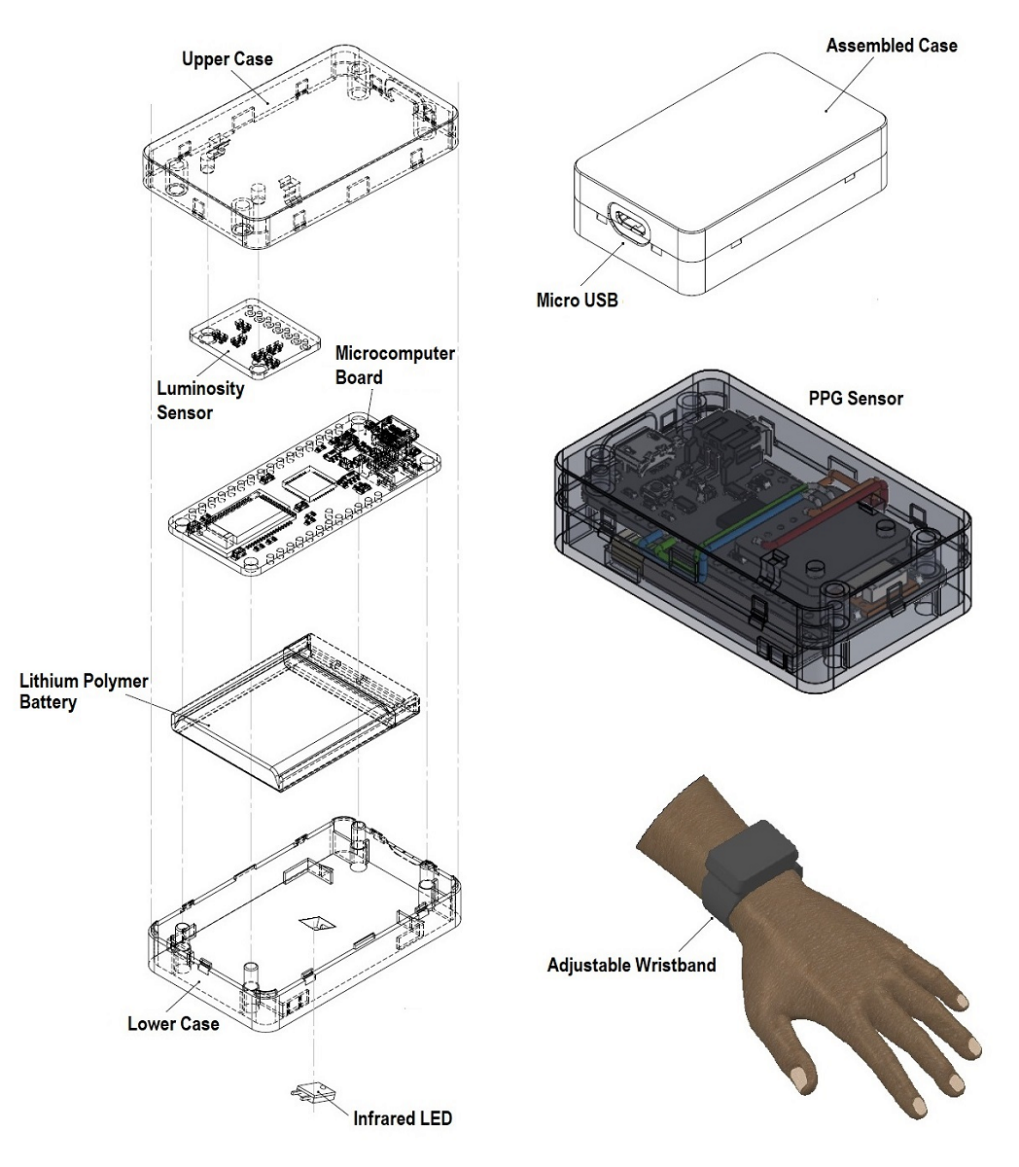
Modular design of the photoplethysmography sensor. LED: light-emitting diode; PPG: photoplethysmography.

The modular development of the sensor was performed on an Arduino-compatible microcomputer board from Adafruit [47], which includes an ATmega32u4 portable microcontroller clocked at 8 MHz, a Bluetooth Low Energy (BLE) [48] module, a connector for a lithium polymer battery, and a built-in micro-USB jack for power charging. A TSL2561 luminosity sensor (Adafruit Industries) [49], an infrared LED LTE-302 (Lite-On) with a wavelength of 940 nm, and a rechargeable lithium polymer battery (3.7 V, 500 mAh) were added to the development board. The electronic circuit was embedded in a 3D-printed case and attached to an adjustable wristband. Figure 2 shows the modular design of our PPG sensor.

### Estimation of BP Parameters

BP was estimated using the principle of the oscillometric method that determines SBP and DBP from the value of the mean arterial pressure (MAP), which is assumed to be nearly equal to the maximum oscillation of the cuff pressure applied to an arterial segment [50]. On the other hand, MAP is also estimated as the maximum vascular unloading detected by the PPG signal [51,52]. Therefore, we estimated BP through a combinatorial ANN model in the following two stages: (1) direct estimation of MAP from raw PPG data and (2) feedback of MAP output to estimate SBP and DBP approximations.

In addition, our approach calculated the variability of continuous BP estimations using the average real variability index [53], which provides superior predictive value over conventional BP variability estimators [54]. This method focuses on changes occurring over short time intervals, and thus, it corrects some of the limitations of SD, which only reflects the dispersion of BP measures around the mean. The average real variability index calculates the average of absolute changes between consecutive BP readings as follows:

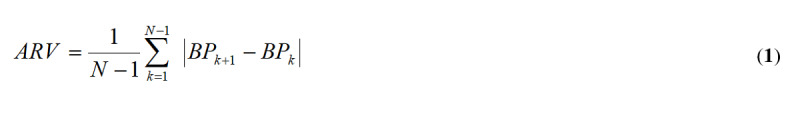



where *N* denotes the number of valid BP measures and *k* is the order of the measures.

### Data Collection Procedure

Fit data for MAP→BP estimation were obtained from the Maracaibo Aging Study [55], which is an ongoing population-based longitudinal study that includes participants aged 55 years or above, who underwent 24-hour ambulatory BP monitoring for the follow-up of cardiovascular outcomes. BP measures were recorded with a validated fully automatic SpaceLabs 90207 device [56], which was programmed to register ambulatory BP readings at 15-minute intervals during the daytime (6:00 AM-10:59 PM) and 30-minute intervals at night (11:00 PM-05:59 AM). We selected subjects with 24-hour BP recordings of good technical quality (>70% valid readings) and obtained a data set of 43,552 BP records belonging to 662 participants (mean age 67.3 years, SD 7.9 years; 69% women). The mean 24-hour BP values were as follows: SBP, 131.1 (SD 21.4) mmHg; DBP, 74.9 (SD 14.4) mmHg; and MAP, 93.6 (SD 15.3) mmHg. The ethical review board of the Institute of Cardiovascular Diseases of the University of Zulia approved the protocol. Written informed consent was obtained from each subject or a close family member.

To collect data for the PPG→MAP problem and test PPG sensor usability, we performed a development and usability study with three older adults (mean age 61.3 years, SD 1.5 years; 66% women) without a history of cardiovascular disease or hypertension. The protocol consisted of individual sessions involving three rounds (8:00 AM-12:00 PM, 2:00 PM-6:00 PM, and 8:00 PM-10:00 PM) to record simultaneous measures with our PPG sensor and a validated semiautomated upper-arm cuff oscillometric device (Omron HEM-4030; BP accuracy ±3 mmHg) [57]. Each round included 10 minutes of rest before starting to acquire measures from participants sitting in the upright position with the back supported, legs uncrossed, and nondominant arm on a desk. BP and PPG readings were taken every 5 minutes by a trained observer until obtaining a specific number of valid records for each round (20, 20, and 10 for the three rounds). The nondominant arm of each participant was equipped with the oscillometric cuff device and PPG sensor placed on top of the wrist with moderate pressure. The BP reference device required a maximum of 30 seconds to detect SBP and DBP. During each BP acquisition, the PPG sensor was programed to collect light absorption readings every 0.6 seconds; values greater than zero were saved and averaged to obtain one PPG measurement for each reference BP measure. The corresponding MAP value was calculated as follows:

*MAP* = (2 × *DBP* + *SBP*) / 3 (**2**)

The fit data set contained a total of 150 values (three participants × one session × 50 measures/session) for MAP (mean 88.9 mmHg, SD 16.1 mmHg) and PPG (mean 4100.7 a.u., SD 107.7 a.u.) measurements. The data acquisition procedure was performed in a private indoor room maintained at a controlled temperature of 25°C and supervised by medical staff. Written informed consent was obtained from all participants. They were compensated US $50 per session, and the protocol was approved by the ethical review board of the Polytechnic University of Sinaloa.

### Machine Learning Model

The ANN model was built using the Neural Network Fitting App from Matlab [58]. Data fitting problems (PPG→MAP and MAP→BP) were addressed by separate two-layer feed-forward networks. In both cases, we used sigmoid and linear transfer functions for the hidden and output layers, and the Bayesian regularization backpropagation algorithm with random weights and bias initialization as the training function. Each fit data set was divided into three sets randomly (70%, 15%, and 15%) for training, validation, and testing. The performance of network outputs with respect to targets was assessed using linear regression analysis. In order to avoid overfitting, training stopped when we reached 1000 epochs or the validation error failed to decrease for six iterations.

### Software Development

We used Matlab Compiler SDK [59] to deploy the trained combinatorial ANN model in a smartphone. The mobile app to display BP estimates from network output was developed in the integrated development environment Android Studio [60]. Communication with the wearable sensor to acquire input PPG data was enabled through the Android Bluetooth serial port profile library [61]. This connection was exclusive, because a BLE peripheral can be paired to only one central device at a time. Android multithreading [62] allowed the smartphone to maintain normal operations while receiving real-time PPG signals. The smartphone app included time setting parameters for the start of monitoring and the measurement frequency of continuous BP readings, and a battery power indicator for the PPG sensor. To provide a user-friendly app for elderly subjects with reduced vision and manual dexterity, we used a simplified graphical user interface with a bright screen, large text, and numbers, and simple input buttons with touchscreen technology, all of which have been proven to be suitable for older users [63].

### Performance Assessment of BP Estimation

To assess performance, we compared outputs of the PPG→MAP→BP combinatorial ANN model (n=150) against BP reference measures recorded with the oscillometric device. The accuracy and precision of comparisons were evaluated through mean error (ME) (SD), Pearson and Spearman correlations, and percentage accuracy. The ME was calculated as the average of differences between reference and predicted BP values. For percentage accuracy, we quantified the error proportion as the absolute difference between reference and predicted values, with division by the reference BP value. We then subtracted this result from 1 and multiplied it by 100. Thereafter, we calculated the mean across all results to obtain the percentage accuracy for BP estimation [42]. Additionally, we assessed BP prediction according to the following: (1) cumulative percentage of absolute differences between reference and predicted values within 5, 10, and 15 mmHg [36]; (2) maximum error of 5 mmHg and 8 mmHg for ME and SD values, respectively [45]; and (3) agreement between reference and predicted values by a Bland-Altman plot [64].

## Results

### PPG Sensor Device

**Figure 3:**
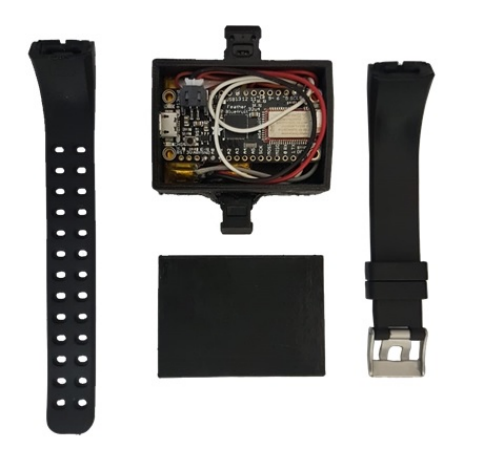
Prototype of the self-designed photoplethysmography sensor device.

The prototype of the PPG sensor is shown in Figure 3. The 3D-printed case (black PLA printer filament) containing the electronic circuit measured 55 mm × 40 mm × 15 mm and weighed 35 g. The distance between the light source and the luminosity sensor was 3 mm. Power consumption ranged from 0.066 W to 0.231 W for the passive and active modes. The circumference range for the adjustable wristband attached to the case was 160 to 240 mm. The cost of all electronic components was US $32.

### ANN Performance for Fitting Problems

**Figure 4:**
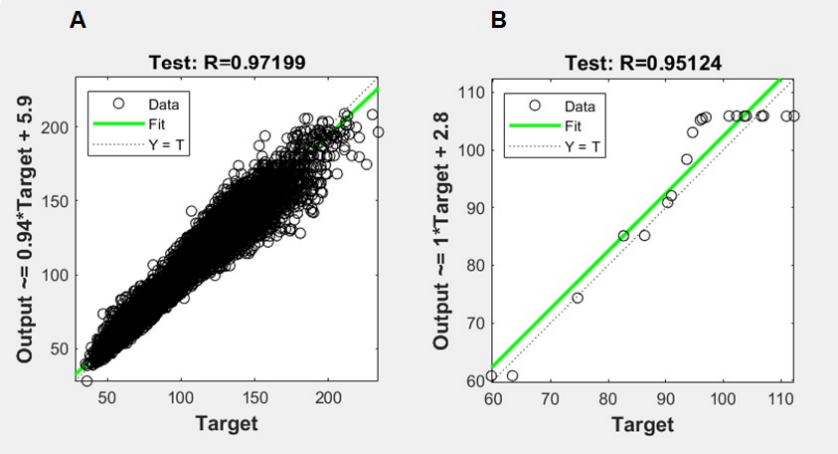
Regression plots for the test set of (A) mean arterial pressure→blood pressure and (B) photoplethysmography→mean arterial pressure fitting artificial neural network models.

Fit data sets corresponding to MAP→BP (n=43,552) and PPG→MAP (n=150) problems were divided respectively into 30,486, 6553, and 6553 and 104, 23, and 23 samples for training, validation, and testing. The best performance for network outputs according to regression analysis was reached using 30 and 10 hidden neurons to separately map the BP and MAP targets. Figure 4 shows regression plots displaying the correlation of network outputs with respect to targets for each test set of MAP→BP (R=0.97) and PPG→MAP (R=0.95) fitting ANN models.

### Accuracy and Precision of BP Prediction

**Figure 5:**
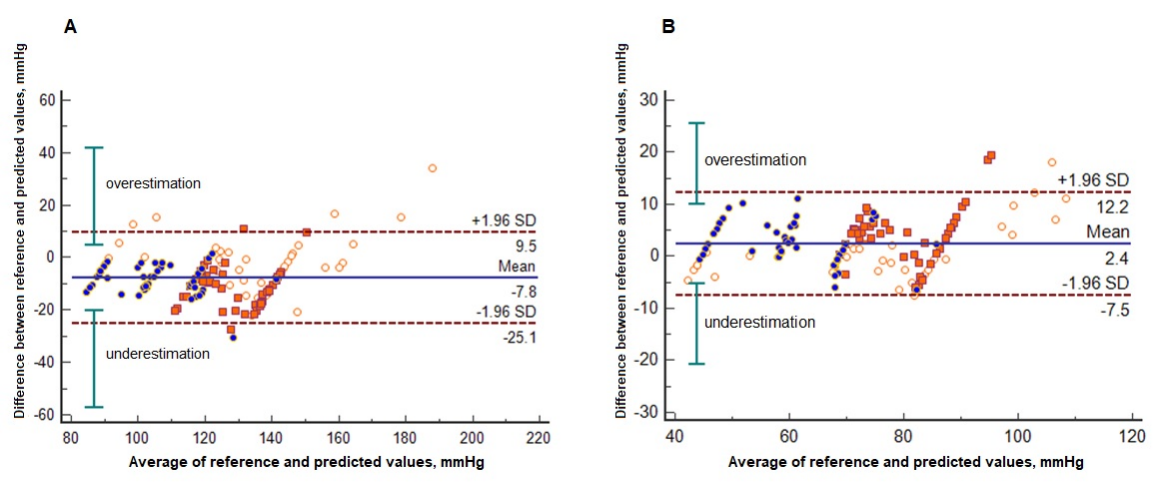
Bland-Altman plots with multiple (A) systolic blood pressure and (B) diastolic blood pressure measures per subject.

Table 2 shows the performance of our combinatorial ANN model in terms of predicting all BP measures recorded with the oscillometric device. The mean error bias (SD) was −7.77 (8.58) mmHg for SBP and −1.02 (4.21) mmHg for DBP. Network outputs predicted SBP and DBP with an average accuracy of 91.72% and 96.67%, respectively. Pearson and Spearman coefficients indicated a very strong correlation (>0.90, *P*<.001) between prediction and BP measurements. The percentages of all predicted values that absolutely differed from reference BP measures by 5, 10, and 15 mmHg or less were 25.33% (38/150), 54.67% (82/150), and 81.33% (122/150) for SBP and 76% (114/150), 98.67% (148/150), and 99.33% (149/150) for DBP. The average of DBP predictions fell within the tolerable error of 5 (SD 8) mmHg, and SBP predictions were close to this accuracy threshold. Overestimation and underestimation of all predicted values with respect to reference BP measures according to a Bland-Altman plot are shown in Figure 5. Table 3 shows the prediction assessment for each participant.

**Table 2:** Accuracy and precision of predicted values with respect to reference blood pressure measures.

BP^a^ estimate	Error bias (mmHg), mean (SD)	Accuracy (%)	Correlation coefficient	
			Pearson	Spearman
SBP^b^	−7.77 (8.58)	91.72	0.91^c^	0.94^c^
DBP^d^	−1.02 (4.21)	96.67	0.97^c^	0.98^c^

^a^BP: blood pressure.

^b^SBP: systolic blood pressure.

^c^*P*<.001.

^d^DBP: diastolic blood pressure.

**Table 3:** Assessment of blood pressure prediction for each participant.

Participant	Error bias (mmHg), mean (SD)		Accuracy (%)	
	SBP^a^	DBP^b^	SBP	DBP
A	−3.75 (10.34)	−0.93 (5.29)	93.58	96.03
B	−11.24 (7.59)	−1.21 (3.98)	90.07	97.01
C	−8.32 (5.51)	−0.91 (3.16)	91.51	96.97

^a^SBP: systolic blood pressure.

^b^DBP: diastolic blood pressure.

### Mobile App Interface

**Figure 6:**
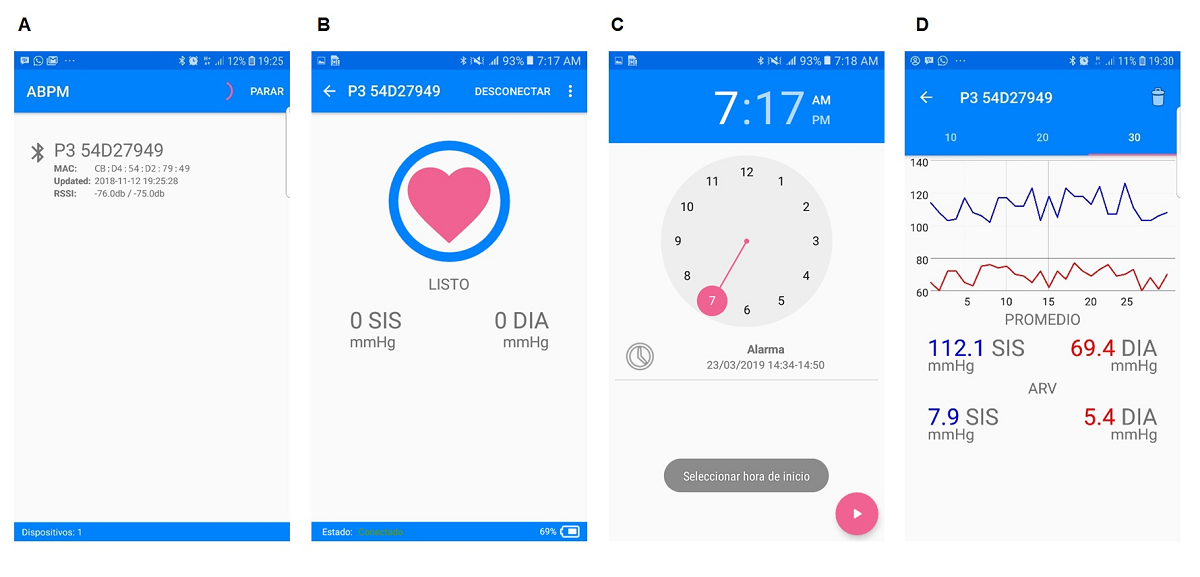
Screenshots of the mobile app operations for (A) Bluetooth pairing between the smartphone and photoplethysmography (PPG) sensor, (B) established connection showing the battery power indicator of the PPG sensor, (C) setting of the start time for blood pressure (BP) monitoring, and (D) deployment of continuous BP measures, average BP level, and BP variability estimation.

Screenshots of the interface of the developed mobile app running on a smartphone using Android operating system are depicted in Figure 6. We present the following four basic operations: pairing the smartphone with the PPG sensor through BLE, the smartphone ready to acquire PPG signals, setting the start time for BP monitoring, and deployment of the BP estimates.

## Discussion

### Principal Findings

Most PPG approaches to estimate BP use pulse transit time calculation, a feature extraction stage, and PPG signals reflected from finger vascular tone [12,14,17]. However, the nonlinearity of the blood volume-pressure relation indicates that the robust performance of these PPG-based BP monitors depends on specific sensing and biological parameters, such as signal recording of high quality, and individual physical property valuation. Therefore, obtaining acceptable BP estimation requires a filtering process to mitigate the effects of contaminated signals by voluntary or involuntary body movements and a calibration step to identify personal cardiovascular and respiratory factors that vary the propagated PPG signal from living tissue.

On the other hand, the portability and location of PPG sensors are key factors to continuously, comfortably, and accurately monitor the BP of users during normal daily activities. In this sense, it is necessary to reduce the number of biosensors for minimizing invasiveness and operational complexity, to avoid PPG measurement from obtrusive body locations that may restrict user movement, and to diminish ambient light influence or displacement of a sensor that can affect PPG signal quality.

To overcome these issues, we proposed a smartphone PPG-only approach for continuous BP level and variability estimation using raw PPG signals, without a calibration procedure. In order to address nonlinear, complex, and dynamic relationships between blood volumetric pulsations and BP changes of the artery, we initially analyzed the correlation between PPG and MAP readings [51,65] for identifying mutual information that can be used to derive BP approximation, instead of examining correspondence on morphological features of PPG and BP waveforms. Furthermore, we developed a small, light, convenient, and wearable PPG sensor wrapped with moderate pressure around the wrist, with the goal of optimizing user comfort and reducing the effects of motion artifacts or inaccurate measurement of PPG signals caused by poorly estimated finger pressing against the PPG sensor [13,39,66].

The proposed combinatorial ANN model showed good accuracy in terms of predicting the reference BP values of our validation sample. On average, our approach predicted SBP and DBP measures with accuracy over 90% and correlations over 0.90 (*P*<.001). Bland-Altman plots showed that most of the errors for BP prediction were less than 10 mmHg. According to the AAMI standard, predicted values successfully fell within the reference measurements for DBP estimation and were very close to the established limit for SBP estimation. In reference to the BHS protocol, the prediction of DBP consistently reached the best accuracy grade, whereas that of SBP reached approximately the boundary accuracy grade. In agreement with previous studies, the error bias range for DBP was narrower than that for SBP in reference to the AAMI and BHS criteria, likely because the variability of DBP is regularly less than that of SBP [17]. On the other hand, another smartphone-based BP monitor that also estimates the variability of BP has not been reported at the moment.

### Limitations

The main limitation of our study is the relatively small size of the validation cohort (n=3). However, we increased the dimension of the final data set by collecting 50 PPG and MAP measures per subject. Moreover, the key stage for approach accuracy was BP mapping from MAP values, which was sustained by an extensive database with more than 43,500 records (n=662).

Second, our selection strategy of only normotensive participants avoided achieving an even distribution of different BP values. Nevertheless, the heterogeneous BP measures recorded by participants (Figure 5) helped cover a wider range of BP values to mitigate part of this limitation.

Third, the development and usability study focused only on estimating BP measures when individuals were in a calm state and under controlled conditions. Therefore, for effective assessment of continuous BP monitoring, it is necessary to validate our findings by upgrading the record of BP estimates during everyday activities in different environments.

Fourth, we included only older subjects for training, validation, and testing of our machine learning approach, and consequently, PPG signals were very susceptible to vascular aging. Since it is one of the factors that can lead to arterial stiffness [67,68], this could represent an overfitting problem to accurately estimate BP in the general population. Nevertheless, one of the major advantages of ANN models is their generalization ability for fitting outputs from new inputs. On the other hand, we mainly addressed continuous monitoring of BP in older adults because they represent a high-risk population for hypertension development.

Finally, despite the potential correspondence between MAP and PPG signals, this relationship may be more complex than previously anticipated [69]. Therefore, future approaches, including additional input user parameters, such as age, gender, and body mass index, may be required to develop a more robust ANN model for BP estimation.

### Conclusion

In this paper, a novel smartphone-based BP monitor was proposed for noninvasive, pervasive, and continuous BP level and variability estimation, using a cuffless, calibration-free, wireless, and wearable PPG-only sensor. We addressed the nonlinear relationship between BP and PPG signals through a multilayer perceptron machine learning approach for estimating BP from raw PPG signals and without a conventional feature extraction stage. The findings of this development and usability study indicated that the employed ANN model performed with good average accuracy and very strong correlation. However, according to the AAMI and BHS standards, only DBP prediction met the clinically accepted accuracy thresholds. Therefore, with further development and validation in real-life conditions, the developed mobile personal health care system could provide a cost-effective strategy for the early diagnosis and control of hypertension and an independent cardiovascular predictor (abnormal BP variability), particularly in rural zones, areas lacking physicians, and areas with solitary elderly populations.

## Abbreviations

AAMI: Association for the Advancement of Medical Instrumentation
ANN: artificial neural network
BHS: British Hypertension Society
BLE: Bluetooth Low Energy
BP: blood pressure
DBP: diastolic blood pressure
LED: light-emitting diode
MAP: mean arterial pressure
PPG: photoplethysmography
SBP: systolic blood pressure
